# Is wearing high heels a female mating strategy? Revisiting the original study using qualitative methods

**DOI:** 10.3389/fpsyg.2022.938916

**Published:** 2022-10-05

**Authors:** Radomír Masaryk, Nikola Synak, Michaela Belišová

**Affiliations:** Institute of Applied Psychology, Faculty of Social and Economic Sciences, Comenius University Bratislava, Bratislava, Slovakia

**Keywords:** physical attractiveness, dating, beautification, partner selection, sexual communication

## Abstract

The 2020 study entitled ‘Wearing high heels as female mating strategy’ by Pavol Prokop and Jana Švancárová claimed that when females imagined an interaction with an attractive male, their preference for high heels steeply increased, compared with an imagined interaction with an unattractive male. The authors concluded that wearing high heels seem to be a form of sexual signaling by females in intersexual interactions. The present paper revisits this study through a psychological standpoint, rather than a biological one. In addition to proposing hypothetical dating scenarios, as in the original study, we also asked participants about how they went about getting ready to go on a date, the significance of dating to them, and their thinking behind choosing particular outfits for a date. We conducted ten focus groups (*N* = 50), recruiting from a similar sample of participants to those in the original study. For our study we followed principles of Thematic Analysis to identify the key themes in the narratives related to dating and beautification. We also used the photo elicitation methodology to observe what footwear our participants own. Our data interpretation from these two sources suggests that young women tend to see dates as social events not necessarily leading to sex; that they do not regard high heel shoes as a means of beautification; and that they take account of practical considerations when getting dressed up for a date. Moreover, young women tend to use beautification with caution. We conclude that the relationship between the tendency to use beautification and attractiveness of a potential partner is far from straightforward; and relying on binary responses to hypothetical scenarios does not provide convincing evidence.

## Introduction

In their paper, Prokop and Švancárová (2020) claim that wearing high heels operates as a form of sexual signaling by females in intersexual interactions. They observed that when female participants imagined an interaction with an attractive male, their preference for high heels steeply increased. However, the study design is based on participants imagining having an interaction with one of two different photographs (attractive/unattractive mele) and choosing one of two kinds of footwear (as shown on a photographs (high heels and low heels). Our study was designed to open up alternative explanations, based on psychological, rather than biological approaches. We used qualitative methods to explore how young women view dating events, with focusing in particular on themes associated with the phenomenon of beautification. Our intention was to explore the narratives of similar young women (*N* = 21 years, SD = 4.67 in the original study) that relate to dating and beautification. Our aim was to gain deeper insights beyond the simple forced choice response used in the original study.

The theoretical foundation of the original paper (Prokop and Švancárová, 2020) is based on the premise that *“human females invest in offspring more than males”* (supported by references to Eibl-Eibesfeldt, 1989; Hewlett, 1992; Geary, 2000) and *“human males, on the other hand, spend more time caring for their offspring than males of virtually all other mammals”* (Bjorklund and Shackelford, 1999; Puts, 2010). According to these sources, parental investment constitutes a basis for female competition over a potential sexual partner. One of the ways for females to achieve this according to Prokop and Švancárová (2020) is beautification, as exemplified by wearing high heels. The parental investment theory (Trivers, 1972) has however, been repeatedly criticized by psychologists on several grounds. Buss (1994) claims that people show a remarkable measure of creativity when it comes to their reproduction strategies. One example is the model of a single long-term relationship combined with several concurring extra-marital affairs. His study involving 10,047 respondents from 37 cultures (Buss, 1989, 1994) provides evidence that women choose their potential partner based on the potential access of men to resources and their willingness to share these resources, as well as their capability to physically protect the family and aspects such as compatibility of their objectives and values. Most importantly, the authors demonstrate that sexual strategies in humans may be complex and multi-faceted.

The association between wearing high heels and sexuality has nevertheless been well documented by several studies. For instance, Morris et al. (2013) found that participants viewed women wearing high heel shoes on point-light videos as significantly more attractive than those with flat shoes. The authors attribute this to increased femininity of gait (reduced stride length and increased rotation and tilt of the hips) and conclude that a woman walking in high heels is a supernormal stimulus. Similar findings were reported in a more recent study by Wade et al. (2022): silhouettes showing women wearing high heels were rated as more attractive, both physically and sexually than the silhouettes of women in flat shoes. The high heel wearers were also seen as more feminine and as of higher status. The 2016 systematic review by Barnish et al. (2018) also confirmed the association between wearing high heels and increased attractiveness and/or an impact on men’s behavior. However, they also confirm the well-documented association between wearing high heel wear and negative musculoskeletal health effects. Wade et al. (2022) suggest that the pain and damage is a trade-off for an increase in perceived attractiveness. High heels were also found to be heavily represented in pornographic imagery (Dietz and Evans, 1982), which may suggest that they have become a cultural symbol of sexuality.

Target groups of the studies in question typically focus on female college undergraduate students. Although the college environment is often stereotyped as highly sexualized, the American College Health Association (2018) reported that 32.3% of female college students have never engaged in oral sex and 33.9% of female college students have never engaged in vaginal sex. Hills (2015) ironically states that *“college campuses are portrayed as being this hotbed of hookup culture“;* the author however, claims that it is not necessarily always the case and a significant proportion of college students are much less sexually active than portrayed in popular culture and media. This leads to a question whether reproductive success or sexual pleasure are truly the driving forces behind clothing and footwear decisions for this target group.

Although the relationship between wearing high heels and physical attractiveness has been repeatedly documented, little is known about the wider context of decisions made by those who decide to wear them. Studies of this kind typically used videos, photographs, silhouettes, or point-light videos in order to reduce the situation to a binary choice of wearing high heels or flats. The study by Prokop and Švancárová (2020) used a hypothetical scenario. This raises several methodological issues. For example, one person looked submissive, introverted, shy, and visibly shorter, while the other seemed much more dominant, extroverted, self-confident, and taller. Forced choice between the two images may yield an answer in keeping with social stereotypes but may not indicate real preference. When McDaniel (2005) asked if there is support for the popular myth that young women prefer jerks as dating partners rather than nice guys, she concluded that young women choose to date nice guys when they are perceived as possessing a combination of attractive personality traits. Physical attractiveness is only one of the factors involved in addition to other qualities such as being funny/witty, romantic, exciting, and someone whom their friends might like. Based on this we believe young women in a hypothetical scenario based their decision on imagined qualities and personality traits of young men in the images rather than on perceived physical attractiveness. Moreover, as one of the reviewers of the present paper suggested in anonymous peer-review, the hypothetical of what would a woman wear to a date with someone they find unattractive is very questionable.

The authors of the original paper suggest that when choosing footwear, a shorter body height is one of the factors (Prokop and Švancárová, 2020). We agree that this could be one of many different factors that come into play when young women choose their outfit for a date. Such decisions are complex and should not be reduced to potential sexual interest. While typical studies in the field attempt to clear the choice of its context, our intention was exactly the opposite: try to examine the choice of footwear within the wider context of dating decisions. The objective of the present paper was to look deeper into narratives expressed by young women both generally as well as specifically when presented with the choice offered in the original paper by Prokop and Švancárová (2020).

## Materials and methods

### Research sample

The research sample consisted of 50 women aged 19 to 24 years (*M* = 21.04; SD = 3.25), which was similar to the sample in the original study (*M* = 21 years, SD = 4.67). Our sample was recruited among students at five universities in Slovakia and one in Czechia. Participants were recruited using university social networks and awarded a small extra credit for coursework. Most of the participants studied humanities, especially psychology (94%). Out of the total number of participants, 34 (68%) reported being in a relationship. The mean number of previous sexual partners ranged from 0 to 13 (*M* = 2.90; SD = 2.47). The mean height of our participants was 168.61 cm or 5.53 ft (SD = 40.29).

### Data collection

Having received ethical approval from the local Institutional Review Board we conducted 10 focus groups consisting of 4 to 6 participants each. Participants who agreed to participate in the study were given a chance to choose from possible time slots to their schedule until we reached the maximum number of 6 for each focus group. Groups were facilitated by the two women researchers who co-authored the present study, to avoid embarrassment while discussing sensitive issues.

In advance of every group, we sent our participants registration forms requesting their contact information, education level, and partnership status. We also asked them to provide a photograph of their entire shoe collection available at the current place of residence (solicited photography). The pictures were used during focus groups to aid memory recall. After each group we sent them a questionnaire with further questions such as age, place of residence, height, the number of shoes and the number of high-heel shoes in their possession, frequency of wearing high-heel shoes, the number of romantic and sexual partners. We asked them to evaluate their own level of attractiveness on a scale of 1 to 10, and to evaluate the level of attractiveness of the two men in the pictures used in the original study by Prokop and Švancárová (2020). We conduced focus groups in October and November 2020 during the Covid-19 pandemic. Due to government measures applicable at that time we had to conduct focus groups online using Microsoft Teams software.

### Original pictures used as stimuli

With the permission of the authors of the original study Prokop and Švancárová (2020), we used their pictures of two men as stimuli, asking our participants what their first thoughts would be if the man in question had invited them on a date. We also asked about their general opinion of both men, and what they would wear to a date with him. The original authors found these pictures online, they were intended to represent an unattractive and an attractive man, respectively.

### Data analysis

We used Thematic Analysis to analyze our qualitative data and followed the process suggested in their methodological paper titled “Using thematic analysis in psychology,” and developed the approach in more depth in their 2021 book. The authors describe Thematic Analysis as a method for systematically identifying, organizing, and offering insight into patterns of meaning (themes) across a dataset. This method allows the researcher to see and make sense of collective or shared meanings and experiences. We chose this method because it allows for flexibility, permits the summarization of key properties in a dataset, and can generate unexpected knowledge. We recorded all of the focus group narratives and created transcripts from them. In the first stage we sought to identify the main themes relevant to our research questions. We then coded all the material and structured the codes hierarchically using Atlas.ti software: we assigned a code to every relevant unit of meaning that summarized its content, then we grouped coded into themes. The procedure observed all relevant ethical guidelines and identities of our subjects were not revealed.

## Results

Our analysis identified four main themes in narratives regarding preparation for going on a date: (1) Young women see dates primarily as social events, rather than leading to sex; (2) High heels are not the primary means of beautification for young women; (3) When dressing up for a date, young women consider practical aspects; and (4) Young women use beautification with caution.

### Young women see dating as social events not necessarily leading to sex

When discussing dating it is vital to understand what participants understand under the term “date”, where would this happen, how intimate such events could become. More than 52% of participants preferred dating in places such as restaurants, bars, cafes – often saying they felt safer in public spaces. When asked about a typical date, 46.2% would prefer going out (dinner, a wine bar, a beer pub; the legal age for drinking alcoholic beverages is 18 in Slovakia), and a further 28.8% envisaged a date as a walk or a trip. Associations were not primarily sexual; dates were more likely associated with enjoyable conversation and socialization. A date was generally considered to be a social event; here are two typical examples of describing dates:

*V1: “We went to a restaurant, ordered dinner and just talked, it was all very cute.”*

*E1: “I associate my typical date with a dinner or a coffee and good conversation.”*

When asked directly if they would consider having sex on a first date, 62% claimed they could not even imagine it. Participants refused sex on the first date mainly because they needed to get to know the partner and establish trust:

*N1: “I cannot even imagine it; I need to get to know the person better and I would not feel comfortable if it happened.”*

A further 36% explained that they could imagine having sex on the first date under certain circumstances, emphasizing that it would depend on how the date developed.

Overall, it seems that young women see first dates more as social events and opportunities to “screen out” potential partners. The idea of having sex on the first date seems to be remote to this age group. About a third admits it could happen, but only under the right circumstances.

### High heels are not the primary means of beautification for young women

Although high heels may be associated with sexuality and seduction in popular culture or pornography, young women do not seem to share this association. We analyzed associations and most of our participants (59.1%) associated high-heel footwear with social events, 12.1% with work or school, 12.1% with dating, 9.1% with informal socialization such as going out for a coffee or dancing, and 7.6% would wear them to any suitable occasion. As many as 39 out of our 50 participants reported only wearing high heels on special occasions, and these were almost exclusively formal events such as weddings, prom nights, formal celebrations, family events, even religious functions. Here is a typical response regarding the events when they had last worn their high-heel shoes:

K4: *“I choose high heels mostly for events such as a family celebration, an anniversary, a wedding, a holy communion, simply whenever I have to dress up.”*

**FIGURE 1:**
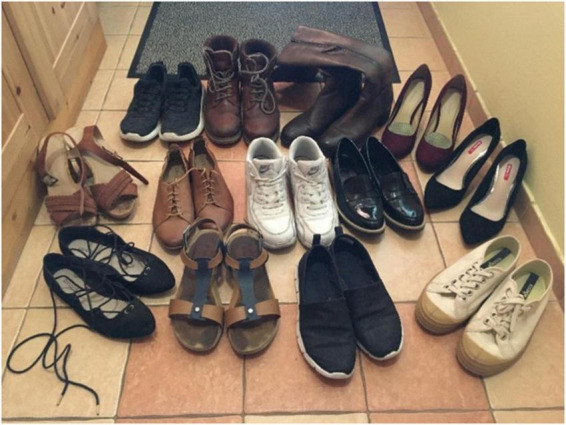
Photograph of a shoe collection, Participant M1.

As an example, Participant M1 provided a photograph (see Figure 1) which suggests that her shoe collection contains two pairs of high heel shoes. The black high heel shoes on the right were only worn twice; she bought them for a Christmas party at her workplace. The other pair of high heel shoes (purple) were only worn at a prom night several years previously. All her remaining pairs are flat, four pairs are sneakers. This photograph represents a typical shoe collection of a young woman in our sample.

Young women in our sample generally preferred sneakers as their footwear of choice. Some specific brands such as Vans or Converse were associated with prestige or value.

We asked about beautification using this phrasing: *“Imagine a person you have fancied for some time asks you to go out on a Friday night. What would you wear? What would be going on in your head when choosing your outfit?”* Participants mentioned applying make-up, using jewelry (necklace, earrings), curling their hair – but only 8 answers included high heel shoes as spontaneous association.

To sum up, the majority of young women in our sample do not wear high heel shoes on a regular basis, only on special occasions. The only exceptions were those who wore such footwear professionally or semi-professionally – such as professional dancers or models. Our sample included two young women who tend to wear high heels regularly.

### When dressing up for a date, young women consider practical aspects

When dressing up for a date young women tend to make themselves more beautiful, but the decision-making process includes very practical issues. When asked *“How do you decide what to wear to a romantic date?”* our participants named a lot of practical things they usually consider, and here are the most frequent ones:

(1) **Location of the date** (32 answers): Do they ride public transport to get there? Will there be cobblestone paved streets to walk through? Is there a chance of an outdoorsy walk or a hike?
(2) **Weather** (25 answers): Temperature, expected rain- or snowfall, etc.
(3) **Coordination with the partner** (15 answers): The style of the partner, and in case of high heels, also the height difference.

In summary, when choosing high heels or flats most of the young women consider location, weather, and coordination with partner.

### Young women use beautification with caution

Our focus group participants do not always tend to beatify, they often do the very opposite – they try to avoid undesirable attention from men and dress down for situations where they have little control over interactions (e.g., when using public transport), or when they expect a company of people other than their peers and friends. Even in the context of dating they may try to project a casual image or divert attention from their bodies. When asked *“How would you dress for a date with Man 1, if he asked you out on a date?”*, 22.6% of the participants said they would not think too much about their clothes, and the most frequent answer was: jeans. With the more attractive Man 2, 19.3% would wear jeans, so there was very little difference (although 38.8% expressed they would put more thought and effort into their choice of outfit for a date with Man 2). Mean level of attractiveness of Man 1 was reported as *M* = 2.88 (SD = 2.23) while the attractiveness of Man 2 was *M* = 7.5 (SD = 3.73).

For example, our Participant K2 told us that on her first dates she tends not to show too much skin and tries to dress casually until she finds out more about the man and his intentions regarding a potential relationship. Another participant, L2 described jeans as “nicer clothes” when describing a hypothetical date with the “attractive” gentleman on the photograph:

*“I would dress up nicer, differently than with the previous guy*. (…) *I would put on blue jeans and a nicer t-shirt or a blouse with ornaments, something more special. And sneakers in summer and boots with flat heels in winter.”*

It seems that young women do use beautification consciously, but make sure to practice caution to avoid coming across as too eager or attracting undesirable attention to their bodies.

## Discussion

As pointed out by Skipper and Nass (1966), dating fulfils a range of different functions. Respondents in our focus groups did not see dates as events leading to eventual reproduction, none of them expressed their desire to start a family anytime in the foreseeable future. They also did not see a date as an event that is necessarily linked with sex. The dating behavior of young women in our sample seemed more in line with observations by McDaniel (2005) – they searched for a combination of attractiveness and other positive personality traits. Although they seem to employ “behavioral tactics” (cf. Prokop and Švancárová, 2020) such as beautification, they use it with caution to avoid appearing too eager or projecting the wrong image.

Although high heels may be associated with sexuality in the pornographic imagery (Dietz and Evans, 1982), our respondents do not appear to use them as a common means of beautification. High heel shoes are much more associated with formal events and the need to comply with social norms. Moreover, only two out of 50 participants wear high heel shoes regularly, similarly to Prokop and Švancárová (2020) who reported that only two women in their sample of 292 young women claimed to wear high-heeled shoes eleven to twelve times per month and only one reported wearing shoes with high heels more than 25 times per month. Out of our 50 participants, 59.1% associate high-heel footwear with social events and reported wearing high heels on formal occasions such as weddings, prom nights, celebrations, etc. Although Wade and al. suggest high heels may trigger expectations of higher status, our participants would be more likely to wear sneakers of premium brands to demonstrate status in informal setting. Moreover, they would consider a range of other factors when selecting their outfit and footwear, primarily the location and associated practical issues, secondly the weather, and thirdly, coordination with their partner, which also seems to be a significant factor in determining the choice of outfit. This confirms the observation by Barnish et al. (2018) who pointed out that no study assessed the role of the respective heights of men and women in this regard: many of our participants mentioned the height of the man as one of the important factors when deciding about the height of their heels.

Our focus group interviews suggest that young women do not seek a father of their future children when dating. Dating is mostly thought of as a social activity and does not necessarily lead to sex. Moreover, young women in our focus groups never brought up the topic of reproduction; having children is something that should be done after finishing college. In Slovakia this generally means three years of Bachelor training and 2 years of training for a Master?s degree. The participants in the original study as well as in our study were mostly still in the early years of their Bachelor studies.

There is a range of psychology studies that disprove the notion of rationality of partner choice. The summary of conceptualizations of love (Masaryk, 2012) suggests different neurobiological processes for sexual desire (mediated by gonadal estrogens and androgens) and for romantic love (mediated by reward systems such as endogenous opioids, catecholamines and neuropeptides such as oxytocin). There are, however, many other elements that may come into play. For example, one of the most cited authors in this regard, Robert Sternberg and Barnes (1988), later added a narrative component to his triangular theory of love. Couples whose narratives are complementary (not necessarily matching!) are the ones who tend to report satisfaction with their relationships. Another example would be Helen Fisher (2009) taxonomy of four groups (Explorers, Builders, Directors, Negotiators) tested on a sample of 7 million questionnaires. These studies suggest that partner choice is much more complicated than just psychical attractiveness.

When we introduced the two images to replicate the hypothetical scenario, the women asked the interviewers many additional questions to get a better understanding of the person. A mere photographic image appeared not to contain enough information for them to make a choice regarding the attractiveness of the man. Although they understood that one of the partners appeared to correspond to the cultural standard of attractiveness more than the other, they were still not sure whether he would make a suitable partner for a date. Many of our participants feared that the “attractive” man would be too intimidating, too promiscuous, unwilling to seek commitment, or simply not the kind of person with whom they would feel comfortable.

Our findings suggest that introducing a hypothetical scenario with two different young men does not necessarily lead young women to choose one as the preferred sexual partner. Although they may give such answer in a forced choice questionnaire, they usually hesitated to answer it in our focus groups and asked several questions to more fully understand the context of the date and the background of the man. As explained above, they seemed to understand the appeal of the “attractive” man, and understood this would be the culturally approved choice of the two, but they expressed many negative associations with the person in the picture. On the other hand, the “unattractive” man was viewed by some participants as cute, more successful, introverted, and less dominant. He was clearly not seen as someone who would be automatically disqualified from being a dating choice based on his appearance. They admitted they would wear nicer clothes to a date with Man 2, but we argue this would be more of an attempt to match his higher perceived social status than an attempt to beautify themselves in order to increase their mating chances. Although the authors had the images rated by fourteen women volunteers (who were older than the sample, at the mean age *M* = 25; SD = 1.74), our participants did not see the men as two extreme poles on the binary scale of attractive/unattractive. Nevertheless, they understood that one of them was more dominant and taller, and the other more submissive and shorter. Hence, if pressed, they would give answers corresponding to social convention. All this, on the other hand, does not entirely disprove the notion that high heel shoes are a strong sexual signal – but it seems they clearly do not serve this purpose for this age group of college undergraduates. Research involving older women with different experiences or from more deorived backgrounds might produce different results. A limitation of our study would obviously be the sensitive nature of the topic of dating and sexuality. Although we tried to create a very safe and trustful environment with two female researchers who were only marginally older than the participants, there is definitely a certain level of social desirability involved. We nevertheless believe that our study brings a more in-depth level of scrutiny, as well as more subtle and nuanced interpretation to the issues involved.

## Conclusion

In conclusion, greater sophistication is needed beyond accepting simplistic assumptions about the way that women are driven to seek the most attractive partner to increase their reproductive chances. Humans clearly do not engage in sex solely to reproduce, and they do not go on dates only to have sex. Beautification or putting effort into choosing clothes may not necessarily relate to the drive for reproductive success and it may not be in any way linked to engaging in sexual intercourse. We argue, therefore, that using evolutionary theories as the basis for research on human behavior is not useful; and we advocate a shift to theories based on psychological, sociological and cultural factors.

Our qualitative take on the issue chosen by Prokop and Švancárová (2020) casts real doubt on the assertion that wearing high heels is a female mating strategy. Even in their own research, 38% of women reported never wearing high heels, 45% reported wearing them once per month, and the remaining 17% reported wearing them more than two times per month. This alone would question the notion of wearing high heels being a regular part of dating behavior. Our data using photo solicitations suggests these numbers may be greatly exaggerated and young collegiate women in this age group wear high heel shoes far less frequently.

It is clear that young women do use beautification when preparing for a date. Specific forms however are likely going to be highly culture-specific and largely depend on the socio-economic status, cultural expectations, and identification with sub-cultures or fashion trends. Our sample and that collected by Prokop and Švancárová (2020) were from a Central European setting; it would be interesting to compare this with samples from other regions and look into possible relations with cultural values such as masculinity vs. femininity (cf. Bašnáková et al., 2016).

More and better research is needed to gain insight into decision-making by young people when it comes to dating; such research should, however, respect the diversity of social and cultural mores that influence the subtle choices being made. The enormous complexity of social behavior in humans makes it problematic to apply the rigid rules observed in animal mating, and concepts like parental investment may not be applicable to humans in the era when control over biological consequences is regarded as social norm while the imperative to reproduce does not justify breaking this norm. We need to be more attentive to social and psychological aspects when studying human behavior and avoid using science to reify stereotypes which do not accurately portray the reality of everyday life.

## Data availability statement

The datasets presented in this article are not readily available because of ethical and privacy restrictions. Requests to access the datasets should be directed to the corresponding author.

## Ethics statement

The studies involving human participants were reviewed and approved by Local Institutional Review Board, Faculty of Social and Economic Sciences, Comenius University Bratislava. The patients/participants provided their recorded informed consent to participate in this study. Written informed consent was obtained from the individual(s) for the publication of any potentially identifiable images or data included in this article.

## Author contributions

RM designed the study. NS and MB collected data. All authors provided substantial contribution to analyzing and interpreting data for the work, drafting the work, and provided approval for publication of the content.
